# Prevention of CMV/EBV reactivation by double-specific T cells in patients after allogeneic stem cell transplantation: results from the randomized phase I/IIa MULTIVIR-01 study

**DOI:** 10.3389/fimmu.2023.1251593

**Published:** 2023-10-30

**Authors:** Armin Gerbitz, Regina Gary, Michael Aigner, Andreas Moosmann, Anita Kremer, Christoph Schmid, Klaus Hirschbuehl, Eva Wagner, Beate Hauptrock, Daniel Teschner, Wolf Roesler, Bernd Spriewald, Johanna Tischer, Stephanie Moi, Heidi Balzer, Stefanie Schaffer, Judith Bausenwein, Anja Wagner, Franziska Schmidt, Jens Brestrich, Barbara Ullrich, Stefanie Maas, Susanne Herold, Julian Strobel, Robert Zimmermann, Volker Weisbach, Leo Hansmann, Fernanda Lammoglia-Cobo, Mats Remberger, Matthias Stelljes, Francis Ayuk, Robert Zeiser, Andreas Mackensen

**Affiliations:** ^1^ Department of Medicine 5 Hematology/Oncology, University Hospital Erlangen, Erlangen, Germany; ^2^ Princess Margaret Cancer Centre, Division of Medical Oncology/Hematology, Toronto, ON, Canada; ^3^ Department of Medicine 3, LMU University Hospital, Munich, Germany; ^4^ Helmholtz Center Munich, Institute of Virology, Munich, Germany; ^5^ Deutsches Zentrum für Infektionsforschung (DZIF) – German Center for Infection Research, Munich, Germany; ^6^ Department of Medicine 2, University Hospital Augsburg, Augsburg, Germany; ^7^ Department of Medicine 3, University Hospital Mainz, Mainz, Germany; ^8^ Department of Hematology, Oncology and Tumor Immunology, Charite University Hospital Berlin, Berlin, Germany; ^9^ Medical Center for Information and Communication Technology, University Hospital Erlangen, Erlangen, Germany; ^10^ Center for Clinical Studies (CCS), University Hospital Erlangen, Erlangen, Germany; ^11^ Department of Transfusion Medicine, University Hospital Erlangen, Erlangen, Germany; ^12^ Department of Medical Sciences, Uppsala University and Clinical Research and Development Unit (KFUE), Uppsala University Hospital, Uppsala, Sweden; ^13^ Department of Hematology/Oncology, University Hospital Muenster, Muenster, Germany; ^14^ Department of Stem Cell Transplantation, University Hospital Eppendorf, Hamburg, Germany; ^15^ Department of Medicine 1, University Hospital Freiburg, Freiburg, Germany

**Keywords:** cytomegalovirus CMV, Epstein-Barr virus EBV, allogeneic, stem cell transplantation (SCT), epitope specificity, prevention, reactivation

## Abstract

**Introduction:**

Allogeneic stem cell transplantation is used to cure hematologic malignancies or deficiencies of the hematopoietic system. It is associated with severe immunodeficiency of the host early after transplant and therefore early reactivation of latent herpesviruses such as CMV and EBV within the first 100 days are frequent. Small studies and case series indicated that application of herpes virus specific T cells can control and prevent disease in this patient population.

**Methods:**

We report the results of a randomized controlled multi centre phase I/IIa study (MULTIVIR-01) using a newly developed T cell product with specificity for CMV and EBV derived from the allogeneic stem cell grafts used for transplantation. The study aimed at prevention and preemptive treatment of both viruses in patients after allogeneic stem cell transplantation targeting first infusion on day +30. Primary endpoints were acute transfusion reaction and acute-graft versus-host-disease after infusion of activated T cells.

**Results:**

Thirty-three patients were screened and 9 patients were treated with a total of 25 doses of the T cell product. We show that central manufacturing can be achieved successfully under study conditions and the product can be applied without major side effects. Overall survival, transplant related mortality, cumulative incidence of graft versus host disease and number of severe adverse events were not different between treatment and control groups. Expansion of CMV/EBV specific T cells was observed in a fraction of patients, but overall there was no difference in virus reactivation.

**Discussion:**

Our study results indicate peptide stimulated epitope specific T cells derived from stem cell grafts can be administered safely for prevention and preemptive treatment of reactivation without evidence for induction of acute graft versus host disease.

**Clinical trial registration:**

https://clinicaltrials.gov, identifier NCT02227641.

## Introduction

Allogeneic stem cell transplantation (aSCT) in the adult patient aims at curing malignant diseases or other deficiencies of the hematopoietic system. Aside from graft-versus-host disease (GvHD), reactivation of latent herpesviruses represents a major complication significantly contributing to morbidity and impairment of quality of life. Due to the immunosuppressive nature of the transplant procedure and due to medical immunosuppression, herpesvirus reactivations are frequent and often require toxic antiviral treatments. Especially cytomegalovirus (CMV) and Epstein-Barr virus (EBV) impose a considerable risk for treatment success and are therefore monitored in the peripheral blood by PCR post transplant. Available antiviral drugs are effective for CMV, but come at high toxicity for bone marrow, kidney and other organs (1). For EBV no anti-replicating drug is available and commonly CD20 targeting antibodies such as rituximab are used to deplete EBV-transformed B cells that give rise to post-transplant lymphoproliferative disease (PTLD) or are main reservoirs for EBV (2). All drugs used only suppress viral replication but do not solve the problem of the absence of virus-controlling T cells in the early phase after stem cell transplantation and hence reoccurrence of viruses after treatment cessation is frequent. Furthermore, the impact of these treatments on quality of life is quite substantial as they are usually associated with inpatient hospital stays.

The immune response that controls new CMV and EBV infections is dominated by a strong expansion of CD8+ T cells which are reactive to both latent- and lytic-cycle viral antigens and persist after the infection has been cleared (3, 4). In asymptomatic immunocompetent individuals, EBV antigen-specific T cells can constitute up to 5% of circulating CD8+ T cells and this proportion can increase to up to 80% in infectious mononucleosis (5). Remission of PTLD was observed after transfer of T cells specific for selected latent and lytic antigens (6). After aSCT, patients typically have a profound T cell deficiency and furthermore receive immunosuppressive agents for the prevention of acute GvHD. As a result, CMV reactivates in approximately 30-50% of patients, and EBV in about 30%. Reactivation of both viruses can occur as early as day 20 post transplantation, and is associated with T cell deficiency, showing that T cells have a major role in controlling these infections.

Cellular therapy of both CMV and EBV reactivation after aSCT has been used with convincing results in several studies over the past decades (7). However, T cell manufacturing has been limited to those patients that have a seropositive donor, as *de novo* generation of CMV- or EBV-specific T cells by *in vitro* priming is difficult, and their generation by TCR gene transfer (8) has higher technical and regulatory hurdles; therefore, clinical trials on their use in aSCT have only recently been initiated (9). In cases of seronegative donors third party approaches can be used successfully (10–15). The response rates in pre-emptive treatments of CMV with specific T cells is high (16) and in the case of EBV it has been shown that transfused EBV specific T cells can control PTLD and persist over long periods (17, 18) up to decades (19).

A key factor in preventing reactivation of CMV and EBV after aSCT are sustained anti-viral T cell responses. However, the *in vivo* depletion of T cells depletion for the prevention of acute GvHD, for example by using anti-thymocyte globulin (ATG), eliminates host and graft-derived virus-specific T cells resulting in reactivation of both viruses as early as day 20 post stem cell infusion. This depletion requires an early intervention with specific T cells to reconstitute antiviral immunity. However, there has been a lack of randomized controlled trials (RCTs) on EBV or CMV-specific T cell transfer in aSCT, be it in therapeutic or prophylactic settings, and results of RCTs that were completed have not been published (NCT01077908, NCT01220895).

Here we present results from a randomized phase I/IIa study utilizing a manufacturing protocol for CMV- and EBV-specific T cell for prevention and pre-emptive treatment of reactivation of both viruses in patient after aSCT (20). The manufacturing protocol generates virus-specific T cells by epitope specific peptide stimulation using material from the stem cell graft preparation as a T cell source, thus avoiding a second apheresis and thereby avoiding additional burden to the transplant donor. This multicenter study recruited patients from 2014 to 2019 in 5 centers in Germany.

## Patients and methods

### Study design/protocol

This controlled, randomized, open label, multicenter phase I/IIa trial exploring the safety and efficacy of allogeneic donor-derived peptide-stimulated T cells with specificity for CMV and EBV in a preventative/pre-emptive setting in patients after aSCT was approved by the federal authority Paul Ehrlich Institute (No. 2016/01) and by the Institutional Review Board of the University Hospital Erlangen (306_13 Az). It was conducted in accordance with the ethical principles of the Declaration of Helsinki (ClinicalTrials.gov Identifier: NCT02227641). All patients and donors provided written informed consent.

The major goal of this study was to assess feasibility of manufacturing and safety of the T cell product. The primary endpoint was acute transfusion reaction and acute GvHD as a potential late toxicity of the product due to the transfer of activated T cells acute GvHD. The design of the study is shown in detail in **Supplementary Figure 1**. To assess efficacy of the product patients were randomly allocated to receive T cell transfer or no study-specific treatment. 1:1 randomization was performed by block randomization of blocks of two and four. Application of three doses of 5x10^4^ CD3+ T cells/kg bodyweight of the recipient were planned, the first as early as day +30 after aSCT with a delay of maximum 42 days. Subsequent doses could be administered in 30 to 51 days intervals. There was no dose escalation, since the efficacy of T cell transfer was expected to depend on T cell expansion after transfer, and consecutive doses were considered boosts of the primary dose. The study design allowed for a crossover from treatment into control group when manufacturing of the cell product failed, or quality specifications were not met. Secondary endpoints with regard to efficacy of the investigational medicinal product were occurrence of CMV reactivation, occurrence of EBV reactivation, cumulative dose of Valganciclovir/Ganciclovir applied for the treatment of CMV and cumulative dose of rituximab applied for the treatment of EBV reactivation. To assess the reconstitution of the CMV/EBV specific T cell compartment as secondary endpoint, participants were analyzed at the end of study for numbers of CMV/EBV specific T cells in the peripheral blood. Recruitment and treatment of patients is shown in **Supplementary Figure 2**.

Enrollment and randomization were performed within 28 days prior to stem cell transplantation. Details of the patient population enrolled in this study are shown in **Table 1**. When randomized for the treatment group the collection center was informed and informed consent was obtained from the donor by qualified members of the collection center. The collection center was provided with required materials to obtained the required cell number and serum from the donor used for the manufacturing process. Manufacturing was started on the day of transplantation. Day 30 post aSCT as day of first infusion was targeted. Prior to each transfusion of the T cell product (24-48h) patients were screened for signs of infection and GvHD or the use of >0.5mg/kg bodyweight prednisone therapy. Transfusion of the T cell product was performed inpatient. No additional medication was given prior to transfusion. For transfusion a central or peripheral vein line was used. Transfusion volume was 1ml for the T cell product, and the line was subsequently flushed with 500ml of 0.9% sodium chloride solution. Patient was ECG monitored and vital signs were taken every 15 minutes after transfusion for up to 2h. Patient was monitored overnight and discharged the following morning.

**Table 1:** Summary of patient characteristics.

	Treatment (n=9)		Control (n=14)		p-Value
**Age at Transplantation**	**45.0±12.5**		**44.2±10.2**		**0.871**
**Sex**	**Female** **Male**	**2** **7**	**Female** **Male**	**1** **13**	**0.259**
**Disease**	**ALL**	**0**	**ALL**	**3**	**0.202**
	**AML**	**6**	**AML**	**6**	
	**MDS**	**0**	**MDS**	**1**	
	**NHL**	**1**	**NHL**	**3**	
	**MM**	**2**	**MM**	**0**	
	**MPS**	**0**	**MPS**	**1**	
**Time to Transplant (days)**	**401±257**		**302±254**		**0.376**
**Donor**	**SIB** **MUD**	**5** **4**	**SIB** **MUD**	**6** **8**	**0.680**
**Height (cm)**	**169.4±9.6**		**175.2±9.3**		**0.773**
**Weight (kg)**	**76.6±9.8**		**78.3±11.9**		**0.805**
**BSA**	**1.88±0.16**		**1.93±0.17**		**0.834**
**BMI**	**27.2±3.6**		**25.6±3.5**		**0.833**
**Karnovsky Index**	**100** **90** **80** **70**	**2** **6** **1** **0**	**100** **90** **80** **70**	**4** **7** **2** **1**	**0.792**
**ECOG**	**0** **1** **2** **3**	**5** **4** **0** **0**	**0** **1** **2** **3**	**5** **8** **1** **0**	**0.520**
**HSCT-CI**	**0** **1** **2** **3**	**6** **2** **1** **0**	**0** **1** **2** **3**	**7** **1** **3** **3**	**0.322**
**Blood Group**	**A** **B** **O**	**4** **2** **3**	**A** **B** **O**	**8** **1** **5**	**0.574**
**Diabetes**	**Yes** **No**	**0** **9**	**Yes** **No**	**1** **13**	**0.609**
**COPD**	**Yes** **No**	**0** **9**	**Yes** **No**	**0** **14**	**---**
**Hypertonus**	**Yes** **No**	**2** **7**	**Yes** **No**	**5** **9**	**0.657**
**Donor **					
**Age at Donation**	**45.0±12.6**		**43.2±10.2**		**0.620**
**Sex**	**Female** **Male**	**3** **6**	**Female** **Male**	**1** **13**	**0.260**

There was no statistically significant difference with regard to major factors influencing outcome between the groups. Only patients with 10/10 HLA match were included into the study. ALL, acute lymphoblastic leukemia; AML, acute myeloid leukemia; MDS, myelodysplastic syndrome; NHL, Non-Hodgkin-lymphoma; MM, multiple myeloma; MPS, myeloproliferative syndrome; SIB, sibling donor; MUD, matched unrelated donor; BSA, body surface area; BMI, body mass index; ECOG, Eastern Cooperative Oncology Group performance score; HSCT-CI, hematopoietic stem cell transplantation comorbidity index; COPD, chronic obstructive pulmonary disease. For test of significance Fisher-exact and Chi-Square test was used.

The SAE-Management was independently performed by the Centre for Clinical Studies of the University of Erlangen using VigilanceOne™ database for safety data acquisition and reporting.

### Patient cohort

**Figure 1:**
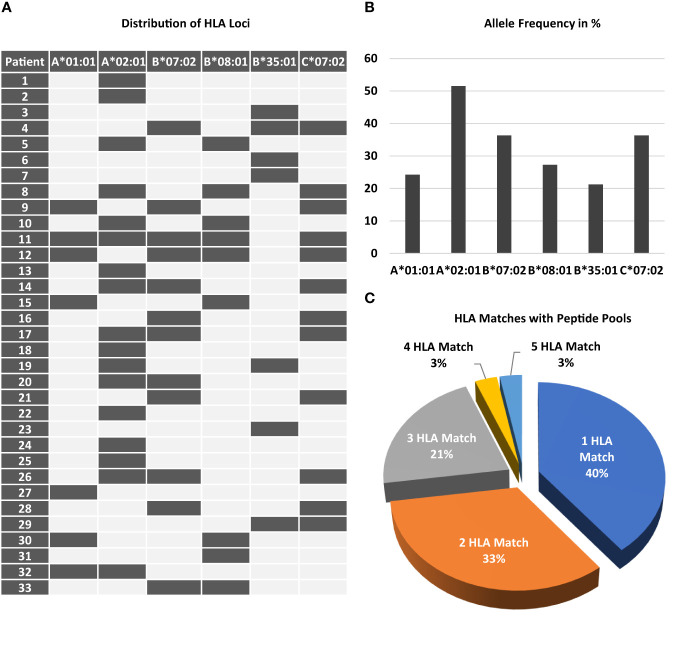
Prevalence of required HLA class I loci among patients screened. Peptides chosen for stimulation required the presence of distinct HLA class I loci. **(A)** The map displays the combination of the 6 different loci and the frequency of each locus **(B)** among the study participant screened. 11 out of 33 patients only had a single HLA class I locus. **(C)** The chart shows the percentage of patients according to the number of HLA matches.

Major inclusion and exclusion criteria are shown in **Supplementary Table 1**. Additional inclusion criteria that had to be met at the time of administration of each dose (pre-screening before treatment) to ensure safety and mitigate risks for the patient (**Supplementary Table 1**). Patient flow during the study period is shown in **Supplementary Figure 2**. Patient characteristics of all patients completing the study are shown in **Table 1**. Prevalence of HLA Loci in the patient cohort is shown in **Figure 1** for all patients screened.

### Manufacturing of T cell product

The manufacturing process (approval number by Government of Oberfranken (DE_BY_05_MIA_2014_0045/55.2-2678.3-6-1) for the T cell product has been described previously (20). Briefly, 1-3x10^9^ G-CSF mobilized PBMC were removed from the G-CSF mobilized peripheral blood allogeneic stem cell graft of CMV and EBV serologically positive donors on the day of collection, representing approximately 3-5% of the entire graft. There was no second apheresis required for the manufacture of this T cell product. Cells could only be drawn from the graft when the graft exceeded 5x10^6^ CD34+ cells/kg bodyweight of the recipient. Upon receipt in the manufacturing unit at University Hospital Erlangen cells were frozen and stored until use. The primary freezing of the raw material was necessary as clean room availability was limited, and immediate initiation of cell manufacture after arrival of cells in the manufacturing unit would not always have been possible. Cells were thawed and stimulated with two peptide pools, one for CMV and one for EBV. Each peptide pool consisted of peptides representing well-characterized epitopes, ten of which were HLA class-I-restricted and seven were HLA class-II-restricted epitopes, as described previously (20) (also see **Table 2**). After 9 days of incubation and expansion, cells were harvested and 3 separate doses of 5x10^4^ CD3+ T cells/kg bodyweight of the patient were cryopreserved. A fourth pilot tube was thawed after the freezing process to test for T-cell viability for each product, as viability above 60% was a release criterion. All products were tested for sterility and the presence of mycoplasma after final cryopreservation. After manufacturing and release of the product, all 3 doses were shipped frozen to the transplant center of the patient and stored locally for subsequent use.

**Table 2:** Peptides used for HLA multimer analysis of virus-specific CD8+ T cells.

CMV No.	Abbr.	Full Sequence	Protein	HLA Restriction	Reference
**1**	VTE	VTEHDTLLY	pp50	A*01:01	Elkington et al. 2003 (21)
**2**	YSE	YSEHPTFTSQY	pp65	A*01:01	Longmate et al. 2001 (22)
**3**	NLV	NLVPMVATV	pp65	A*02:01	Diamond et al. 1997 (23)
**4**	VLE	VLEETSVML	IE-1	A*02:01	Khan et al. 2002 (24)
**5**	TPR	TPRVTGGGAM	pp65	B*07:02	Weekes et al. 1999 (25)
**6**	RPH	RPHERNGFTVL	pp65	B*07:02	Weekes et al. 1999 (25)
**7**	ELK	ELKRKMMYM	IE-1	B*08:01	Elkington et al. 2003 (21)
**8**	QIK	QIKVRVDMV	IE-1	B*08:01	Elkington et al. 2003 (21)
**9**	IPS	IPSINVHHY	pp65	B*35:01	Gavin et al. 1993 (26)
**10**	CRV	CRVLCCYVL	IE-1	C*07:02	Ameres et al. 2013 (27)
EBV No.	Abbr.	Full Sequence	Protein	HLA Restriction	Reference
**1**	CLG	CLGGLLTMV	LMP2	A*02:01	Lee et al. 1993 (28)
**2**	GLC	GLCTLVAML	BMLF1	A*02:01	Steven et al. 1997 (29)
**3**	YVL	YVLDHLIVV	BRLF1	A*02:01	Saulquin et al. 2000 (30)
**4**	FLY	FLYALALLL	LMP2	A*02:01	Meij et al. 2002 (31)
**5**	RPP	RPPIFIRRL	EBNA3A	B*07:02	Hill et al. 1995 (32)
**6**	QAK	QAKWRLQTL	EBNA3A	B*08:01	Burrows et al. 1994 (33)
**7**	RAK	RAKFKQLL	BZLF1	B*08:01	Bogedain et al. 1995 (34)
**8**	YPL	YPLHEQHGM	EBNA3A	B*35:01	Burrows et al. 1994 (33)
**9**	HPV	HPVGEADYFEY	EBNA1	B*35:01	Rickinson et al. 1997 (35)
**10**	EPL	EPLPQGQLTAY	BZLF1	B*35:01	Saulquin et al. 2000 (30)

To assess the presence of CMV/EBV specific CD8+ T cells before and after the T cell product application flow cytometric analysis using peptide loaded HLA multimers was used. The HLA multimers used were selected according to the presence of the HLA genotype of the patient.

### Flow cytometric analysis

All antibodies were purchased from BD Biosciences (Heidelberg, Germany) unless otherwise specified. For phenotypic analysis, cells were stained with anti-CD8 FITC (clone SK1), anti-CD25 PE (clone 2A3), anti-CD14 PerCP (clone MφP9), anti-CD56 APC (clone B159), anti-CD19 PE-Cy7 (clone SJ25C1), anti-CD4 APC-Cy7 (clone RPA-T4), anti-CD3 V450 (clone UCHT1), and anti-CD45 V500 (clone HI30). Gating included the exclusion of debris in a SSC vs. FSC plot. Leukocytes were gated in a CD45 vs SSC dot plot as CD45+ cells. The CD45high/SSClow population was termed lymphocytes and could be distinguished from monocytes and granulocytes. Within CD45high cells, subsets were characterized by expression of CD3 (T-cells), CD19 (B-cells), and CD56 (negative for CD3) NK-cells. Monocytes were gated as CD14+ SSC low cells.

For analysis of CMV- and EBV-specific T-cells, 1x10^6^ PBMC were stained with peptide loaded multimers (ProImmune Ltd., Oxford, United Kingdom) according to manufacturer’s recommendation. **Table 2** shows the peptide and their HLA restriction used for analysis. HLA multimers were used according to the presence of each HLA locus in the patient (see **Figure 1**) and CD8 positive T cells binding HLA multimers were termed CMV or EBV specific respectively. Cells were co-stained with anti-CCR7 FITC (clone 150503, R&D Systems), Fluorotag PE, anti-CD8 PerCP (clone SK1), anti-CD62L APC (clone DREG-56), anti-CD45RA PE-Cy7 (clone HI100), anti-CD4 APC-Cy7 (clone RPA-T4), and anti-CD3 V450 (clone UCHT1). Lymphocytes were gated in the SSC vs FSC dot plot. T cells were defined as CD3+ SSClow. Among T cells, the CD4+ and CD8+ T cells were distinguished. HLA multimer-binding cells were determined as proportion of CD8+ T cells. Cells were analyzed subsequently after staining using a FACS Canto II (Becton Dickinson).

### Statistical analysis

Statistical analysis was performed using IBM SPSS software Version 28. Survival curves were generated using Kaplan-Meyer estimates. Comparison of means was performed using a non parametric Mann-Whitney-U test. To test the frequency distribution of categorical variables a Chi Square test was used. For analysis of contingency tables Fisher exact test was used.

## Results

### Patients and recruitment

Over the study period from 2013 to 2019, 33 patients were screened and 29 patients were enrolled in to the study and randomized (see **Suppl. Figure 2**). Due to block randomization, 16 patients were randomized into the treatment group, while 13 patients were assigned to the control group and were just monitored without any specific treatment. Antiviral treatment could be used according to the centers preference when clinically indicated, including rituximab for EBV reactivation. Within the treatment group, 2 patients crossed over due to missing product release and 1 patient due to refusal of the product. Four patients within the treatment group were excluded from the study before the first application due to medical reasons or withdrawal of consent. In total 9 patients were treated with 25 doses. Two patients did not receive dose 3 due the development of acute GvHD. Of the 9 patients treated, 9 were alive at day 204 post transplantation, the end of study. There was no relapse of the underlying disease in the treatment group. In the control group, 2 of 16 patients relapsed and were excluded from the study in accordance with the study protocol. In total 14 control patients were evaluated at 204 days post transplantation. As shown in **Table 1**, there was no significant difference between treatment and control group with regard to age, disease, donor, and performance scores. Patient average age was 45.0 ± 12.5 in the treatment and 44.2 ± 10.2 years in the control group respectively. Distribution of donor type was not significantly different between the two groups. There was no significant difference regarding the sex of the donor and age of the donor.

Inclusion into the study required the presence of at least one of the following HLA alleles: HLA-A*01:01, A*02:01, B*07:02, B*08:01, B*35:01, or C*07:02. **Figure 1A** shows the distribution and combination of these alleles among all patients randomized. As expected for the population in Germany, HLA-A*02:01 was the most frequent (51.5%) allele (**Figure 1B**). Thirteen of 33 patients (40%) had only one of these alleles, all other patients had 2 or more, one of these had 5 (**Figure 1C**).

### Manufacturing of the T cell product

**Figure 2:**
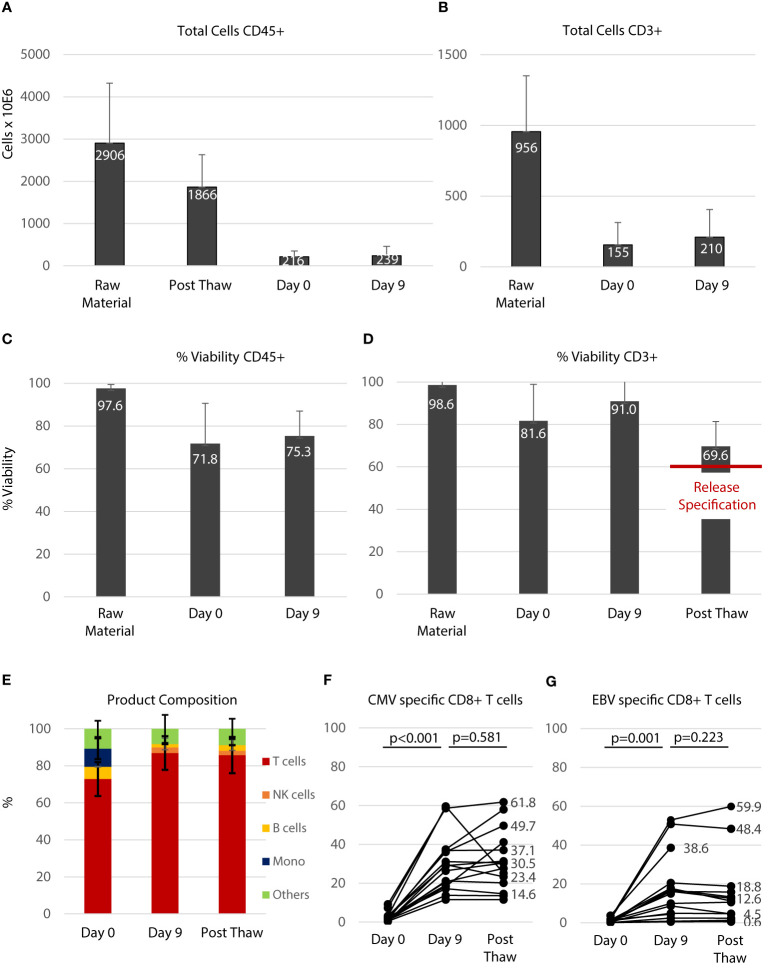
Manufacturing of the T cell product. **(A)** Total number of all leukocytes (CD45+) during the manufacturing process. Raw material indicates the PBMC fraction derived from the stem cell graft. Post Thaw indicates the number after the first cryopreservation process. Day 0 indicates before peptide stimulation and Day 9 indicates the time of cell harvest before final cryopreservation. **(B)** Total number of CD3+ T cells during the manufacturing process. **(C)** Viabilities of CD45+ cells during the manufacturing process in percent. **(D)** Viabilities of CD3+ T cells during the manufacturing process in percent. **(E)** Composition of the T cell product: unstimulated (Day 0), peptide stimulated (Day 9) and after cryopreservation (Post Thaw) which is equivalent to the composition of the final product infused. Numbers **(A–E)** are shown as average ± standard deviation. The percentage of CMV- **(F)** and EBV- **(G)** specific T cells within the CD8+ T-cell fraction was significantly increased during the 9 days of expansion (Day 0 vs. Day 9) and percentage of specific T cells was not affected by cryopreservation (Day 9 vs. Post Thaw, Mann-Whitney-U test).

A total of 17 products were manufactured during the study period. **Table 3** gives an overview of the products and their application. Patient 20 was considered a screening failure and excluded from the study after the product was manufactured. After peptide stimulation and expansion over 9 days, the product was cryopreserved and a pilot tube was analyzed for vitality several days thereafter. Aside from sterility testing, major product release criteria were a sufficient number of CD3+ T cells (acceptable dose range from 2-5x10^4^ CD3+/kg body weight) and a vitality above 60% after thawing of the pilot tube. None of the products manufactured showed bacterial or mycoplasma contamination. The endotoxin concentration in the final product was consistently <500 IU/ml. As shown in **Figure 2A** on average 2,906 ± 1,417 x10^6^ PBMC (raw material) were drawn from the stem cell grafts and used for manufacturing. Approximately one third of the cells were lost in the initial cryopreservation process and 1,866 ± 761x10^6^ cells (Post Thaw) were obtained after thawing. The vast majority of these cells were lost during subsequent washing steps and approximately 7.4% of the cells received from the stem cell graft were used for peptide stimulation (216 ± 136x10^6^, Day 0). The number of CD45-positive cells remained stable over the 9 days of the expansion period (239 ± 219 x10^6^, Day 9). As shown in **Figure 2B**, approximately one third of the cells in the stem cell graft were CD3+ T cells (956 ± 394 x10^6^, Raw Material). On the day of peptide stimulation (Day 0) 155 ± 158x10^6^ live CD3+ T cells were present. This number increased over the 9 days of expansion to 210 ± 195x10^6^ CD3+ T cells. As shown in **Figures 2C, D**, viability in the CD45+ and in the CD3+ compartment was somewhat reduced by the first cryopreservation (compare Raw Material and Day 0). After 9 days of expansion viability was improved, especially in the CD3+ compartment (**Figure 2D**); after cryopreservation of the final product on average 69.6 ± 11.7% of CD3+ cells were viable. The composition of the PBMC fraction at different time points during the manufacturing process is shown in **Figure 2E**. On day 0 at the beginning of the T-cell expansion before peptide stimulation a substantial number of B cells (6.0 ± 4.0%) and monocytes (9.8 ± 5.6%) were present in the PBMC fraction, which played a role in the manufacturing protocol as peptide-presenting cells. On day 9, 85.8 ± 9.8% of all cells were T cells, and the composition of the T cell product was not altered after the final cryopreservation. The specificity of T cells in the T cell product for CMV (**Figure 2F**) and EBV (**Figure 2G**) was assessed by flow cytometry using peptide loaded HLA multimers according to the HLA alleles present (see **Figure 1**) before (Day 0), after peptide stimulation (Day 9), and after final cryopreservation. The proportion of CMV specific CD8+ T cells, calculated as the sum of T cells that stained positive with matched HLA/peptide multimers in each product, was 2.53 ± 2.37% of the PBMC before peptide stimulation and (30.9 ± 15.4%) thereafter (p<0.001). A similar expansion rate was obtained for EBV-specific T cells (day 0: 1.0 ± 0.97%, day 9: 16.3 ± 16.5%, p=0.001). **Supplementary Figures 3** and **4** give an overview on single epitope specificities, T cell subsets and T cell activation in the product generated for patient 11, who had the most HLA matches (See **Figure 1A**). Cryopreservation of the product did not significantly impact the percentage of specific T cells (compare Day 9 and Post Thaw). In summary, central manufacturing of peptide stimulated antigen-specific T cells from G-CSF mobilized stem cell grafts under study conditions is feasible. Approximately 90% of all cells obtained are lost during the manufacturing process resulting in a high percentage of epitope specific T cells not requiring any further selection.

**Table 3:** Overview of T cell products manufactured.

Pat.	CD3 /kg	CD4 /kg	CD8 /kg	CD56 /kg	CD19 /kg	CD3 absolut	Comment
1	18000	n.d.	n.d.	n.d.	n.d.	1,800000	Not applied, product not released, T cell number too low
4	32000	12000	17000	240	1100	2,400000	Applied
5	41000	2300	37000	320	2000	2,500000	Applied
7	23000	9800	10000	590	440	2,450000	Not applied, patient moribund at V2
10	31000	1600	28000	390	450	1,850000	Not applied, patient withdrew consent
11	31000	5100	25000	120	30	2,870000	Applied
13	31000	2800	27000	320	160	3,340000	Not applied, patient refused study medication
16	28000	8200	18000	1700	1700	2,240000	Not applied, product not released, CD3 vitality too low
19	31000	4400	23000	340	480	2,750000	Applied
20	23000	6000	16000	300	250	1,610000	Not applied, screening failure
23	36000	2600	31000	2600	1100	2,240000	Applied, Developed GvHD after 2nd dose
25	36000	1200	35000	37	220	2,560000	Applied, Developed GvHD after 2nd dose
27	33000	1800	29000	1100	140	2,680000	Applied
28	21000	2400	12000	2300	680	1,370000	Applied
29	30000	180	30000	n.d.	n.d.	2,490000	Applied
30	29000	14000	13000	200	67	2,560000	Not applied, screening failure
31	34000	1900	28000	890	140	2,860000	Not applied, patient developed severe skin GvHD before V2

In total 17 products were generated throughout the study. Products transfused are highlighted in dark grey. Light grey indicates products not transfused. The table shows the absolute dosing and dosing/kg bodyweight of the active ingredient (CD3+, possible range 20,000-50,000/kg body weight) and contaminating cells (CD45+, CD19+).

### Application of the T cell product

**Figure 3:**
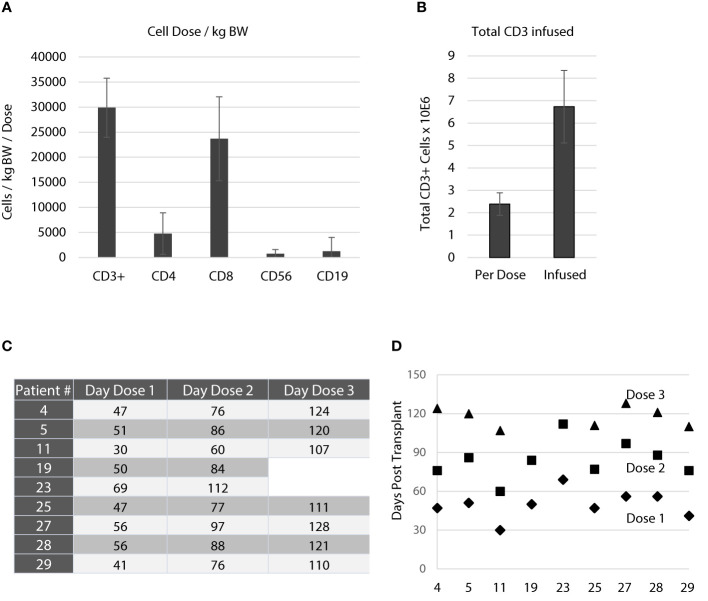
Application of the T cell product. **(A)** Average number of cells/kg bodyweight transfused with each dose. Data are shown as means for all products applied ± standard deviation. The active substance of the T cell product was peptide-stimulated CD3+ cells. The targeted dose was 50,000 CD3+ cells/kg bodyweight/dose. There were only small fractions of contaminations with NK cells (CD56+, 763 ± 812/kg bodyweight/dose) and B cells (CD19+, 1257 ± 2760/kg bodyweight/dose). **(B)** Average total number of CD3+ T cells infused with each dose and average total number of CD3+ T cells infused per patient. Data are shown as means ± standard deviation. **(C)** Three doses were planned throughout the study targeting day 30, day 60 and day 90. The table shows the actual application days for each patient. **(D)** Schematic illustration of the application days for each patient and their deviation from the actual schedule. As indicated in **(C, D)** patient 19 and 23 received only 2 doses.

As this study aimed at prevention and pre-emptive treatment of reactivation of CMV and EBV the dose schedule was planned to start as early as day 30 post aSCT. A total of 3 equal doses was planned for each patient with consecutive doses on days 30, 60, and 90 while patients were still receiving cyclosporine A. The targeted cell dose was 50,000 CD3+ T cells/kg body weight. On average, the cell number per dose administered to patients was 29,882 ± 5,883 CD3+ T cells/kg BW (**Figure 3A**), of which 4,768 ± 4,143 were CD4+ and 23,688 ± 8,372 were CD8+ T cells. On average, a total of 2.39 ± 0.50x10^6^ CD3+ T cells were infused per dose resulting in a total number applied of 6.73 ± 0.50x10^6^ CD3+ T cells (**Figure 3B**). **Table 3** gives a summary of all products manufactured with regard to cell dosing. As shown in **Figure 3C**, the schedule planned for dose administration was not achieved for most patients. On average, the first dose was given 49.8 ± 10.8 days after aSCT. The second (day 84 ± 14.7) and third (day 117.3 ± 8.0) dose were delayed as well. Notably, most of the delays occurred for the first dose and were due to pending sterility data or late start of manufacturing due to clean room constraints. **Figure 3D** graphically illustrates the dosing schedule for each patient. In summary, 7 out of 9 patients received all doses with considerable delay, which was mostly due to technical constraints.

### Adverse events

We observed in total 618 adverse events (AE) in both groups with an average of 24.7 ± 17.2 per patient. As shown in **Table 4**, there was no statistical significance with regard to number or severity (CTC grading) of AEs observed between both groups. Notably, the number of SAE/patient shows a trend toward significance with more SAE in the treatment group (p=0.096). In total we observed 30 severe adverse events in both groups (overall 1.2 ± 1.3/patient), of which 3 were considered severe adverse reactions (SAR) in relation to the T cell product. Patient 19 and 23 developed acute GvHD within 2 to 4 weeks after the second infusion of the T cell product. Patient 23 developed acute GvHD of the gut (overall Glucksberg grade III) requiring frequent hospitalizations. Patient 11 had 4 SAE not directly related to the T cell product. Both, patient 11 (4 SAE) and 23 (5 SAE) received 18 and 55 medical treatments. Both patients together experienced 135 of 244 AE and 9 out of 18 SAE, causing approximately 50% of all events within the treatment group (n=9). Eight of the 23 (35%) patients analyzed did not experience any SAE (n=2 in the treatment group, n=6 in the control group). Number of medical treatments outside of the routine follow up (14.22 ± 6.58 vs. 10.64 ± 5.41, p=0.926) was not significantly different between the two groups. Similarly, the number of hospitalizations (2.89 ± 2.93 vs. 1.71 ± 1.82, p=0.403) was not significantly different between treatment and control group respectively. In summary, there was no difference in terms of adverse events between both groups.

**Table 4:** Events during study.

Event	Treatment (n=9)	Control (n=14)	p-Value
Number of AE/Patient	27.1 ± 24.6	24.4 ± 11.2	0.600
Severity of AE	1.60 ± 0.39	1.64 ± 0.24	0.688
Number of SAE/Patient	2.00 ± 1.73	0.79 ± 0.69	0.096
Number of Medical Treatments/Patient	14.22 ± 6.58	10.64 ± 5.61	0.926
Number of Hospitalisations/Patient	2.89 ± 2.93	1.71 ± 1.82	0.403

Number of AE, SAE, medical treatments, and hospitalizations in both groups. There was no statistical difference between both groups regarding all adverse observations (non-parametric Mann-Whitney-U test).

### Outcomes

**Figure 4:**
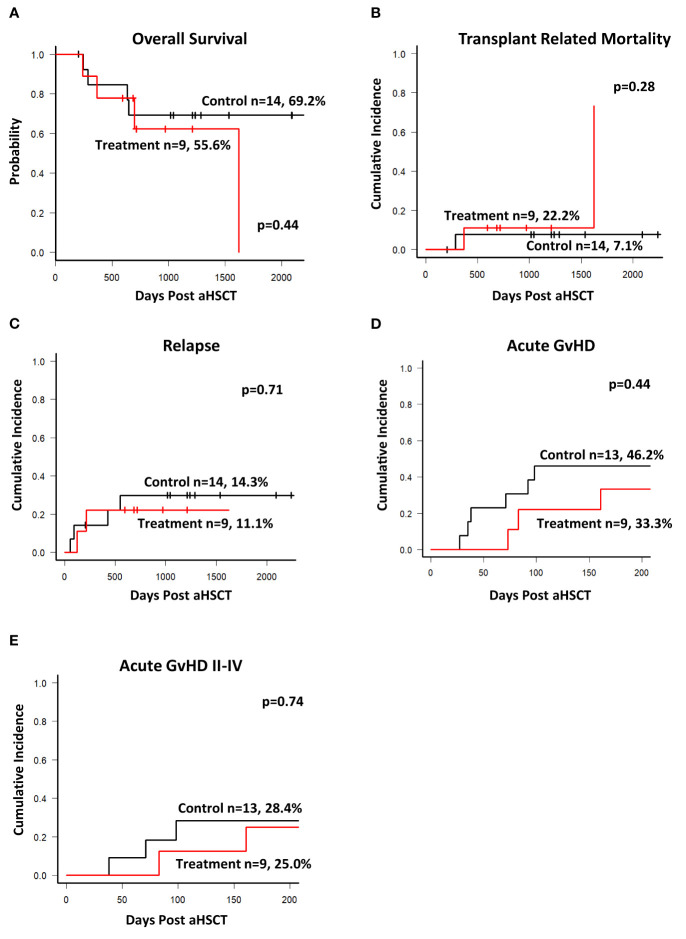
Outcomes. Database was closed on Dec 31^st^ 2020 with a median follow up of 930 days. **(A)** Kaplan-Meier estimates of overall survival of patients in both groups. **(B)** Cumulative incidence of transplant related mortality during the entire observation period. **(C)** Cumulative incidence of Relapse. Cumulative incidence of acute GvHD (all grades, **(D)**) and grade II-IV **(E)** requiring treatment during the study observation period of 204 days. There was no statistically significant difference between the groups for all outcomes shown.

The study recruited patients from 2013 until the end of 2018. The database was closed on 31^st^ of December 2020. The median follow-up was 930 days. As shown in **Figure 4A** overall survival was not significantly different between the two groups. Similarly, we did not observe any difference in the cumulative incidence of transplant related mortality (**Figure 4B**) and relapse of the underlying disease (**Figure 4C**). One patient in the treatment group died on +1623 after aSCT due to a bacterial urosepsis causing the sudden change in survival and TRM curve. There was no evidence that the application of peptide stimulated T cells increased toxicity of the transplant procedure or impacted survival.

### Study endpoints

The primary endpoint of this first-in-human study was toxicity assessed by the incidence of acute transfusion reaction related to the T cell product and/or late toxicity manifested by the development of acute GvHD due to the infusion of activated T cells. As shown previously, the T-cell products can substantially expand in the host and persist long term (18, 20), and a cumulative effect of the three doses is likely. A total of 25 doses of the IMP was applied in 9 patients. There was no acute transfusion reaction and none of the patients developed any signs of cytokine release syndrome or fever within the 24-hour post-infusion observation period. All patients were hemodynamically stable during the first 24 hours post infusion and able to leave the hospital in good condition without any noted side effects. Two patients developed acute GvHD after the second infusion. In one patient (patient 23, **Figure 3C**), this was observed after the second dose on day 86, requiring steroid treatment (overall acute GvHD grade II). In the second patient (patient 24, **Figure 3C**), aGvHD emerged on day 161, 51 days after the second infusion (overall acute GvHD grade III). Both patients were therefore not eligible for receiving the third dose. As shown in **Figures 4D, E**, during the observation period of 204 days post aSCT the cumulative incidence of overall acute GvHD and of acute GvHD grade II-IV requiring treatment was slightly and non-significantly lower in the treatment group than in the control group. In summary, there were no acute events related to the infusion of the T cell product. Overall incidence of acute GvHD was not significantly different between the two groups, and we conclude from these findings that the study met its primary endpoints.

**Figure 5:**
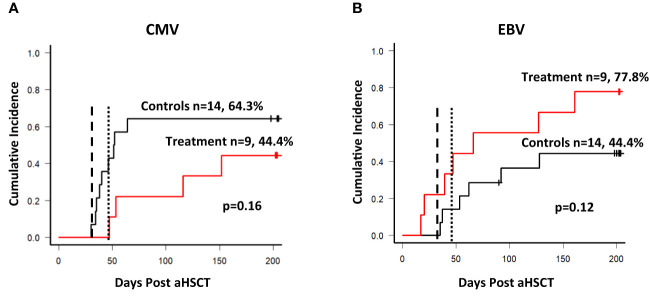
Reactivation of CMV and EBV. **(A)** Cumulative incidence of CMV reactivation among patients. The dashed line in both graphs shows the targeted day of first T cell product infusion (day 30), the dotted line shows the average day of the first infusion (day 49) among the 9 patients. Nine out of 14 patients reactivated CMV within the control arm during the observation period of 204 days, and 4 out of 9 patients in the treatment arm respectively. **(B)** Cumulative incidence of EBV reactivation among patients. Six out of 14 patients reactivated EBV during the observation period, and 7 out of 9 in the treatment arm. The dashed line indicates the targeted day of first infusion, while the dotted line indicates the average day of the first infusion (49).

Secondary endpoints of the study included the incidence of CMV and EBV reactivation, the use of antiviral drugs such as ganciclovir, valganciclovir, foscavir or cidofovir, and the use of rituximab for the treatment of EBV. T-cell reconstitution was assessed in both groups throughout the observation period and was started before the first transfusion of the T cell product. CMV and EBV DNA in peripheral blood plasma or PBMCs was determined by PCR in the laboratory used by the transplant center. Reactivation was defined as a single positive result in a PCR-based test. As shown in **Figure 5**, there was no statistically significant difference in the cumulative incidence of CMV (A) and EBV (B) reactivation between the groups. The earliest CMV reactivation occurred in the control group on day 29, and 5 patients reactivated CMV before day 40. Most CMV reactivations in the control group occurred as expected within the first 100 days post aSCT. When the observation period was limited to the first 100 days the treatment group a significantly lower cumulative incidence of CMV reactivation (p=0.044). In contrast, the earliest EBV reactivations in the treatment group occurred on day 19 and 20, and 5/23 patients reactivated EBV before day 40, three of them in the treatment group. The cumulative incidence of EBV reactivation appeared increased in the treatment group, although not statistically significant (also see **Table 5** for univariate analysis). The T cell products contained B cells as contaminants, although in low numbers (see **Table 3**, CD19), and the possibility of transmitting EBV through administration should not be neglected. However, when the two cases of EBV reactivation prior to the first transfusion of the T cell product were excluded from the analysis this trend disappeared (data not shown) and both curves became superimposed.

**Table 5:** Secondary endpoints.

Event	Treatment (n=9)	Control (n=14)	p-Value
CMV Reactivation	4	9	0.349
Patients treated with Ganciclovir	2	1	0.221
Ganciclovir Treatment Days Mean	20.5±0.7	2.0±0	0.831
Ganciclovir Cumulative Dose Mean mg	4827±1997	600±0	0.831
Patients treated with Valganciclovir	4	6	0.940
Valganciclovir Treatment Days Mean	22.0	37.1	0.212
Valganciclovir Cumulative Dose/Patient mg	32721±18680	26550±20238	0.507
EBV Reactivation	7	6	0.099
Rituximab Doses/Patient	2.5±0.7	1.67±0.6	0.024
Patients treated with Rituximab	2	2	0.580
Rituximab Cumulative Dose/Patient	625.0±216.5	937.5±265.1	0.105

Secondary endpoints included number of patients with CMV and/or EBV reactivation, number of patients treated with Ganciclovir, Valganciclovir, Foscavir, Cidofovir and Rituximab. Cumulative drug dosing was assessed throughout the study for all antiviral drugs including Rituximab. For comparison of events among the groups, Chi-Square test was used. For comparisons of means non-parametric Mann-Whitney-U test was used.

None of the patients received Foscarnet or Cidovovir as a treatment. There was no statistically significant difference in the use of Ganciclovir (3 out of 23 cases). The most frequent drug used for the treatment of CMV was Valganciclovir. There was no difference regarding the number of patients treated, the duration of treatment and cumulative dose applied in each patient (**Table 5**).

### T cell reconstitution

**Figure 6:**
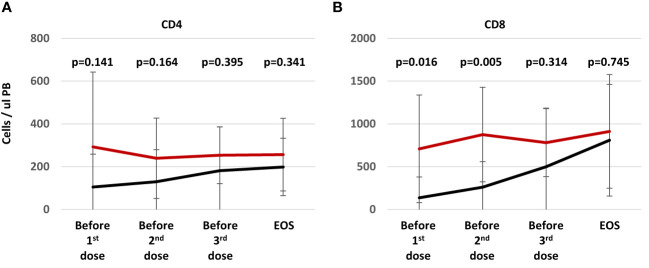
T cell reconstitution during study treatment. Patients (n=9) randomized into treatment group (red line) were analyzed at the time of the visit before the T cell transfer (24-48h, visits 2, 8, and 12). Patients in the control arm (n=13, black line) were analyzed at study visits aligned as possible with the treatment group at day 30 ± 3 (visit 2), 60 ± 3 (visit 8), 90 ± 3 (visit 8), and at the end of study at approximately day 204 ± 7 (visit 17). The graph shows average numbers of CD4+ **(A)** and CD8+ **(B)** T cells per ul peripheral blood ± standard deviation. Statistical differences between both groups were calculated for each time point using a non-parametric Mann-Whitney-U test.

As a secondary endpoint, T cell reconstitution was compared between the two groups at equivalent time points, starting before the first infusion of the T cell product. An example of flow cytometric analysis of peripheral blood is shown in **Supplementary Figure 5**. Numbers of CD4+ T cells (**Figure 6A**) and CD8+ T cells (**Figure 6B**) appeared to be higher in patients of the treatment group at early time points than in the control group. These differences did not reach statistical significance for CD4+ T cells, but for CD8+ T cells numbers were significantly higher before receiving the first or second dose than in controls (**Figure 6B**). Since this difference was present before any T cell infusion, it is impossible to say whether T cell transfer had any effect on overall CD8+ T cell reconstitution. Differences between treatment and control groups became marginal at later time points, when most centers would have begun tapering or ending treatment with cyclosporine A, allowing for better T cell expansion.

**Figure 7:**
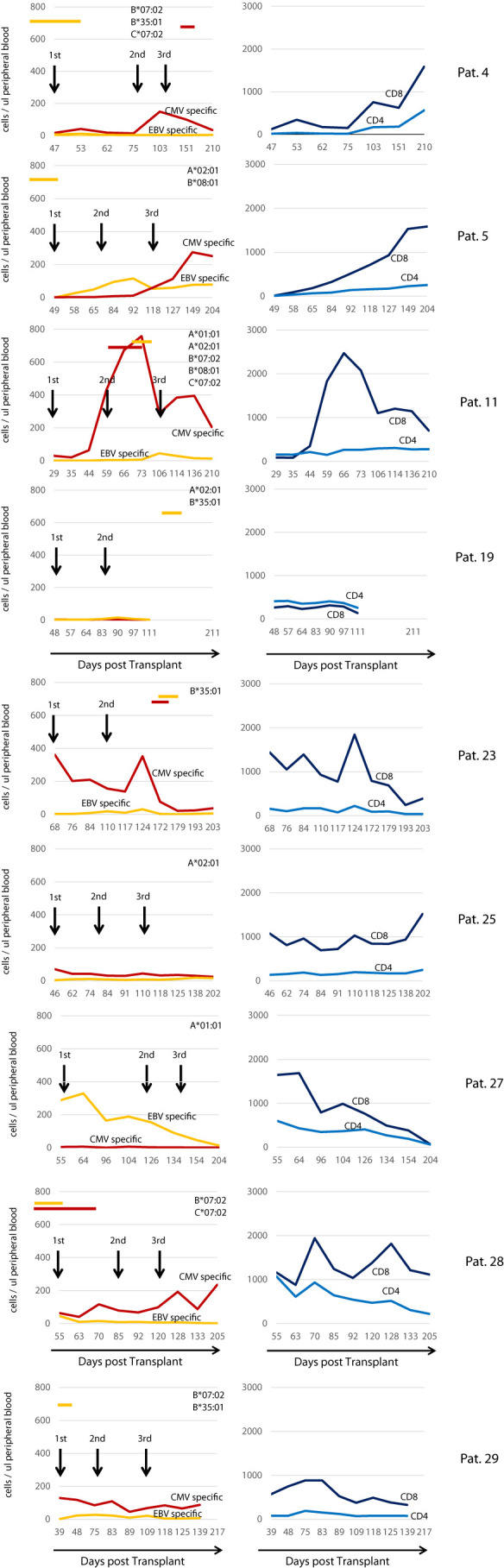
Reconstitution of CMV/EBV specific T cell immunity in treated individuals. Left Panel: Reconstitution of CMV- and EBV-specific T cells during the study observation period. Red lines indicate the sum of all CMV specific in the peripheral blood (cells/ul), yellow lines the sum of all EBV-specific CD8+ T cells detected by using peptide-loaded HLA class I multimers according to the HLA class I loci present in the patient on the top right of each graph. Black arrows indicate the transfusion of the IMP. Red and yellow horizontal bars indicate the presence of CMV and EBV respectively as assessed by PCR in the peripheral blood. Right Panel shows the overall reconstitution (number of cells/ul peripheral blood) of CD4+ (light blue line) and CD8+ (dark blue line) T cells in each individual patient.

**Figure 7** left panel illustrates the reconstitution of CMV- and EBV-specific T cells for all 9 patients having received the T cell product. In a majority of patients, CMV-specific T cells were detected in higher numbers than EBV-specific T cells in peripheral blood. No general pattern was generated with regard to post infusion expansion. In several patients (4, 5, 11, and 28), expansion of CMV- or EBV-specific T cells after some infusions were observed. The highest numbers of CMV-specific T cells were observed in patient 11 between the second and third infusion. Shortly after infusion of the second dose, this patient developed CMV reactivation, which resolved when CMV-specific T cells had reached their peak, and thereafter CMV-specific T cells declined until the third T cell dose was applied. In other patients, there was no clear coincidence of virus-specific T-cell expansion and reactivation of either virus. Notably the time courses of reconstitution of CD8+ T cells correlates in several cases with the expansion of CMV specific T cells (see for example patient: 5, 11, 23, 28). In contrast in patient 27 who did not expand CMV specific T cells, EBV specific T cells correlated with overall CD8+ T cells.

## Discussion

In this paper we present the results of a randomized controlled phase I/IIa study (MULTIVIR-01) aiming at prevention or pre-emptive treatment of CMV/EBV viral reactivation in patients after aSCT. Patients received 3 consecutive doses of 21,000-41,000 CD3+ T cells per kg bodyweight and dose, separated by 2 intervals of at least 30 days. The primary endpoint of the study was to demonstrate safety of the T-cell product as assessed by the occurrence of an acute transfusion reaction and/or as late toxicity the development of acute GvHD. Both endpoints were met, as we did not observe any transfusion reactions in 25 T cell infusions, and the cumulative incidence of acute GvHD was not significantly different between the two groups. Thus, the product can be considered safe for application in humans.

Several studies have aimed at prevention of reactivation predominantly for CMV employing various techniques of manufacturing including selection of specific T cells based on IFNγ production (19), streptamer-based selection technologies (36), and stimulation with cell lines expressing viral antigens or peptide stimulation (20, 37, 38). The challenge herein lies in the early reactivation of CMV and EBV after aSCT especially when *in vivo* T-cell depletion by ATG or Campath is part of the conditioning. Reactivation of both viruses has been shown to happen as early as within the second week after aSCT requiring ready to use readily available T cell products. Our study aimed at 1^st^ infusion on day 30 post aSCT as the manufacturing process took 10 days and safety diagnostics approximately 20 days. However, under real life conditions in our institutions, production required more time than originally envisaged, such that the first product was on average infused on day 49, when one or both viruses had already reactivated in several patients. Delays were mainly due to clean room availability and courier delays. Our study showed that even complex logistical cell manufacturing and product delivery can be achieved centrally, although there is room for improvement of available infrastructures.

To overcome the problem of early reactivation, several studies have employed partially HLA-matched third party CMV-, EBV-, or even multi-virus-specific T cells. In currently ongoing placebo controlled randomized phase III studies this concept is being explored in more than 60 centers worldwide (Clinicaltrials Identifier: NCT05305040) (39). The manufacturing process used in the study presented here could be applied in a similar setting as well, as unused doses of the T cell product could be banked, thereby establishing a third-party library.

The phase II part of this study suffered from low patient numbers in both groups, and we were not able to demonstrate any influence of the T cell product on any of the secondary endpoints monitored. Recruitment into the study was seriously compromised with the marketing authorization of letermovir as a prevention for CMV reactivation in late 2017. No preventative anti-viral treatment other than aciclovir was available when the study started recruiting in 2013. Although funding of letermovir was initially problematic and not secured in Germany, many care takers were reluctant to enroll patients into a phase I study when an approved drug for CMV prevention was available. Hence recruitment slowed down significantly over time and the study stopped recruiting in 2019.

Patient 4, 5, 28 and 29 reactivated EBV as early as day 17, 20, 35 and 39 in the treatment group. These patients received the T-cell product on day 47, 51, 55, and 41, respectively, in a pre-emptive treatment fashion. In all four patients, EBV reactivation resolved within less than 10 days. Patients 4 and 28 were treated for EBV reactivation with 2 doses of rituximab. Our data neither confirm nor exclude a potential role of infused EBV-specific T cells in reversal of EBV reactivation in these patients. Only 2 of 4 patients within this group required treatment with rituximab as the copy numbers observed did not trigger treatment in 2 patients. Similarly, 2 patients of the control group were treated with rituximab; the difference between treatment and control groups was not statistically significant with regard to the use of rituximab. Patients 28 also reactivated CMV before receiving the first infusion of the T-cell product. Patients 4, 11 and 23 reactivated CMV after initiation of T-cell transfer. In contrast, 9 out of 14 patients in the control arm reactivated CMV beginning on day 30. Given this situation and the low number of patients in both groups, the study could not demonstrate prevention of reactivation of either virus. However, we noted a reduction of the cumulative incidence of CMV reactivation in the treatment group, which was significant when the observation period was limited to 100 days.

The function of virus-specific T-cell transfer in prophylaxis and prevention of CMV reactivation or disease in stem cell recipients has been difficult to assess. Several studies have been published over the last two decades (11) and a wide range of efficacy has been reported with regard to reactivation. Similar to our study, Blyth et al. (40, 41) reported a decade ago administration of T cells for prevention of CMV reactivation in aSCT patients. Ex vivo expanded CMV specific T cells were administered on day +28 at a much higher dose level of 2×10^7^/m^2^. The authors did not report any difference in the rate of reactivation of CMV between treatment and control group but reported a significant reduction in peak viral load and subsequently a lower number of patients who required antiviral therapy. However, their study (40) employed T cells that were specific for only one CMV antigen, pp65, or for only one epitope from this antigen, while our present protocol included multi-epitope-specific T cells that targeted epitopes from additional antigens such as CMV IE-1. The capacity of CMV-specific T cells to recognize infected cells varies widely according to their antigen and epitope specificities (27), and IE-1-specific T cells may be more protective in transplantation settings (42, 43). Therefore, different T cell products may be difficult to compare and more comprehensive studies should address the role of antigen and epitope specificity in control of CMV in therapeutic settings in the future.

The study was able to demonstrate expansion of T cells with specificity for CMV and EBV after the infusions in a number of cases. Patients 4, 5, 11, 23 and 28 displayed an increase in T cell numbers after infusion, especially when viral antigens were expected to be present due to reactivation. This is in our view an important finding, as it demonstrates activity of the T cells infused. It is noteworthy that the most substantial increase in T cells was seen in those patients where more than one of the required HLA class I loci was present. Especially presence of HLA B*07:02, B*08:01, and C*07:02 gave rise to a substantial expansion as shown for patient 4, 5, 11, and 28. In the presence of only HLA A*02:01 (patient 25) or in combination with HLA B*35:01 (patient 19) we were not able to demonstrate significant expansion, which is in contrast with our recent findings (18). These findings may reflect dominance of certain HLA restricted epitopes which we were not able to detect when HLA B*07:02 and HLA B*08:01 and C*07:02 were absent. In general, it can be said that there is space for expansion in aSCT patients as early as day 40 which is an important finding of relevance for all early T-cell therapy approaches, including the option of third party derived only partially HLA matched virus specific T cells. While the application of donor derived fully HLA matched virus-specific T cells can be considered safe at this stage, application of only partially HLA matched T cells at early time points bears considerable risk for acute GvHD or even graft rejection. Little is known to date about the fate of these T cells as they are likely to be rejected when the graft is fully established and T-cell chimerism is complete. However, at early time points post aSCT with incomplete T-cell chimerism this may not be the case, especially when presence of viral antigens promote expansion of HLA mismatched T cells as it has been shown that virus-specific T cells can exhibit allo-HLA reactivity (44, 45). Our HLA matched approach would not give rise to such problems.

In summary, we have been able to demonstrate that centralized manufacturing in an academic institution and application of virus specific T cells is feasible in a multi-center setup in spite of high logistical efforts. We have shown that the T-cell product can be safely applied and did not find any toxicity. The study fell short at demonstrating efficacy of the T-cell product mainly due low patient numbers but has provided the foundation for subsequent efficacy studies.

## Data availability statement

The raw data supporting the conclusions of this article will be made available by the authors, without undue reservation.

## Ethics statement

The studies involving humans were approved by Institutional Review Board of the University Hospital Erlangen (306_13 Az). The studies were conducted in accordance with the local legislation and institutional requirements. The participants provided their written informed consent to participate in this study.

## Author contributions

AG: Designed study, wrote study protocol, developed cell product and manufacturing protocol, wrote IB, recruited patients, analyzed data, generated figures, wrote manuscript. RG: Developed manufacturing protocol, wrote IB, performed quality analysis of the cell product, performed manufacturing, analyzed data, generated figures. MA: Designed study, wrote IB, developed manufacturing protocol, wrote manuscript. AMo: Designed study, designed peptide pools, analyzed data, wrote manuscript. AK: Recruited patient as principal investigator at University Hospital of Erlangen, helped analyzing data, helped writing manuscript. CS: Recruited patients as principal investigator at University Hospital of Augsburg. KH: Recruited patients as principal investigator at University Hospital of Augsburg. EW: Recruited patients as principal investigator at University Hospital Mainz. BH: Recruited patients at University Hospital Mainz. DT: Recruited patients at University Hospital Mainz. WR: Recruited patient as principal investigator at University Hospital of Erlangen. BS: Recruited patient as co-principal investigator at University Hospital of Erlangen. JT: Recruited patient as principal investigator at University Hospital of Munich. SMo: Manufactured T cell products, performed quality analysis of products. HB: Manufactured T cell products, helped in quality analysis. SS: Manufactured T cell products, helped in quality analysis. JBa: Manufactured T cell products, helped in quality analysis. AW: Coordinated logistics and communication with donor centers. Organized transportation of products and raw material. FS: Coordinated logistics and communication with donor centers. Organized transportation of products and raw material. Helped consenting patients at University Hospital Erlangen. Applied products. JBr: Recruited and consented patients at Charite University Hospital Berlin. BU: Designed secuTrial ECRF and VigilanceOne™ database. Prepared and analyzed data. SMa: Designed study, helped writing study protocol and IB, communicated with authorities, performed data analysis and submission. SH: Designed study, helped writing study protocol and IB, communicated with authorities, performed data analysis and submission. Performed pharmacovilgilance. JS: Supervised manufacturing and quality analysis, performed product release. RZi: Helped writing IB, supervised manufacturing and quality analysis, performed product release. VW: Supervised manufacturing and quality analysis, performed product release. LH: Recruited patients at Charite University Hospital Berlin, analyzed data, helped writing manuscript. FL-C: Analyzed data, analyzed T cell products, performed patient follow up analysis at Charite University Hospital Berlin. MR: performed statistical analysis, helped analyzing data. MS: Served as member of the Data Safety Monitoring Board. FA: Served as member of the Data Safety Monitoring Board. RZe: Served as member of the Data Safety Monitoring Board. AMa: Designed study, wrote study protocol, recruited patients, analyzed data, wrote manuscript. All authors contributed to the article and approved the submitted version.

## Supplementary material

Supplementary Figure 1 Study design overview. Patients were randomized at visit 1 before allogeneic stem cell transplantation between day -28 and -7. Transplant procedure was carried out according to standards of the participating centers. A small fraction of the stem cell graft (3-5% of all cells) was shipped along with donor serum to the manufacturing site at the University Hospital of Erlangen. Manufacturing was started at the day of transplantation. If the T cell product could not be released or manufacturing failed, patients were able to cross over from the treatment group into the control group. Twenty-four to 48 h before each T cell product application patients were screened for acute GvHD including steroid treatment and signs of infection. If patients were not eligible for T cell product application this visit was repeated as additional visit 7 days later. The time frame for this assessment was 42-days opening a window for the first treatment from day 30 to day 72. Patients in the control group were monitored accordingly. End of study visit was 204 days after allogeneic stem cell transplantation.

Supplementary Figure 2 Study recruitment overview. Schematic overview of the patient flow of all patients screened. In the treatment arm 3 patients crossed over to the control arm, as the product could not be released, or patient refused study medication. Four patients we not considered eligible for the study medication at visit 2 and were excluded from the study. In the control arm 2 patients were excluded due to relapse and the development of post transplant proliferative disease (PTLD).

Supplementary Figure 3 Pat 11, Epitope specific T cell expansion during manufacturing. PBMC derived from the stem cell graft were analyzed before peptide stimulation (Day 0) and after 9 days of expansion (Day 9) by flow cytometry. Patient 11 shared 5 out the 6 HLA types for which peptides from CMV and EBV were available. Cells were gated on CD8+ T cells. Upper panel (red frame) shows specificities of CD8 for CMV epitopes, lower panel for EBV epitopes respectively (yellow frame).

Supplementary Figure 4 Pat 11, T cell subsets and activation during manufacturing. PBMC derived from the stem cell graft were analyzed before peptide stimulation Day 0 and after 9 days of expansion Day 9 by flow cytometry. Cells were gated on CD3+ T cells. **(A)**: T cell subsets analyzed by the expression of CD45RA and CCR7. naïve: CD45RAhigh/CCCR7high, central memory: CD45RAlow/CCR7high, effector and effector memory: CD45RAlow/CCR7low, TEMRA: CD45RAhigh/CCR7low before and after peptide stimulation. **(B)**: Expression of CD25 as T cell activation marker on CD4+ and CD8+ T cells before and after peptide stimulation. Peptide stimulation resulted in expansion of the effector and effector memory compartment, while reducing the naïve and TEMRA compartment. At the end of the expansion period, approximately 41.9% of CD4+ and 57.3% of CD8+ T cells were activated shown by CD25 expression.

Supplementary Figure 5 Flow cytometry of epitope specific T cells after adoptive transfer. Example of flow cytometric assessment of virus specific T cells in the peripheral blood of patient 5, 14 days after the second infusion of the T cell product. The patient was positive for HLA*A02:01 and B*08:01. As shown in four epitopes (CLG, GLC, YVL, FLY) presented on HLA A*02:01 and two epitopes (QAK, RAK) presented on HLA*B08:01 could be used to stimulate donor-derived EBV-specific T cells. Two CMV derived epitopes (NLV, VLE) with restriction to HLA A*02:01 and two epitopes with restriction to HLA B*08:01 were available in the peptide pools to stimulate CMV specific T cells. The same peptides were used to load HLA pentamers for flow cytometric analysis. **(A)** Following lysis of red cells, peripheral blood samples were gated on lymphocytes (FSC, SSC), CD3, CD4 and CD8. **(B)** CD8+ T cells were then analyzed for binding of peptide loaded HLA pentamers. Absolute numbers were calculated using a defined number of beads per volume of blood sample. As shown in this example a 7.43% of all CD8+ T cells showed specificity for 6 EBV derived epitopes and 8.49% were specific for 4 CMV derived epitopes respectively.

Supplementary Table 1 Study Inclusion/Exclusion criteria. Inclusion criteria are shown for patients and donors separately. Only patients with serologic positive (CMV, EBV) 10/10 HLA-matched donors were included in the study. In addition, the study protocol required the presence of at least one of the following HLA class I loci: A*01:01, A*02:01, B*07:02, B*08:01, B*35:01, C*07:02. Before each T cell product transfusion “additional inclusion criteria” were applied as indicated to prevent infusion when acute GvHD or treatment with steroids, or signs of infection are present.
