# Supplementary material for: Prevention of CMV/EBV reactivation by double-specific T cells in patients after allogeneic stem cell transplantation: results from the randomized phase I/IIa MULTIVIR-01 study
Source: Front Immunol. 2023 Oct 30;14:1251593. doi: 10.3389/fimmu.2023.1251593 (PMC10642256; doi:10.3389/fimmu.2023.1251593)
Supplement: Supplementary Figure 1 — Study design overview. Patients were randomized at visit 1 before allogeneic stem cell transplantation between day -28 and -7. Transplant procedure was carried out according to standards of the participating centers. A small fraction of the stem cell graft (3-5% of all cells) was shipped along with donor serum to the manufacturing site at the University Hospital of Erlangen. Manufacturing was started at the day of transplantation. If the T cell product could not be released or manufacturing failed, patients were able to cross over from the treatment group into the control group. Twenty-four to 48 h before each T cell product application patients were screened for acute GvHD including steroid treatment and signs of infection. If patients were not eligible for T cell product application this visit was repeated as additional visit 7 days later. The time frame for this assessment was 42-days opening a window for the first treatment from day 30 to day 72. Patients in the control group were monitored accordingly. End of study visit was 204 days after allogeneic stem cell transplantation. [file Presentation_1.pdf]

Supplementary Figure 1

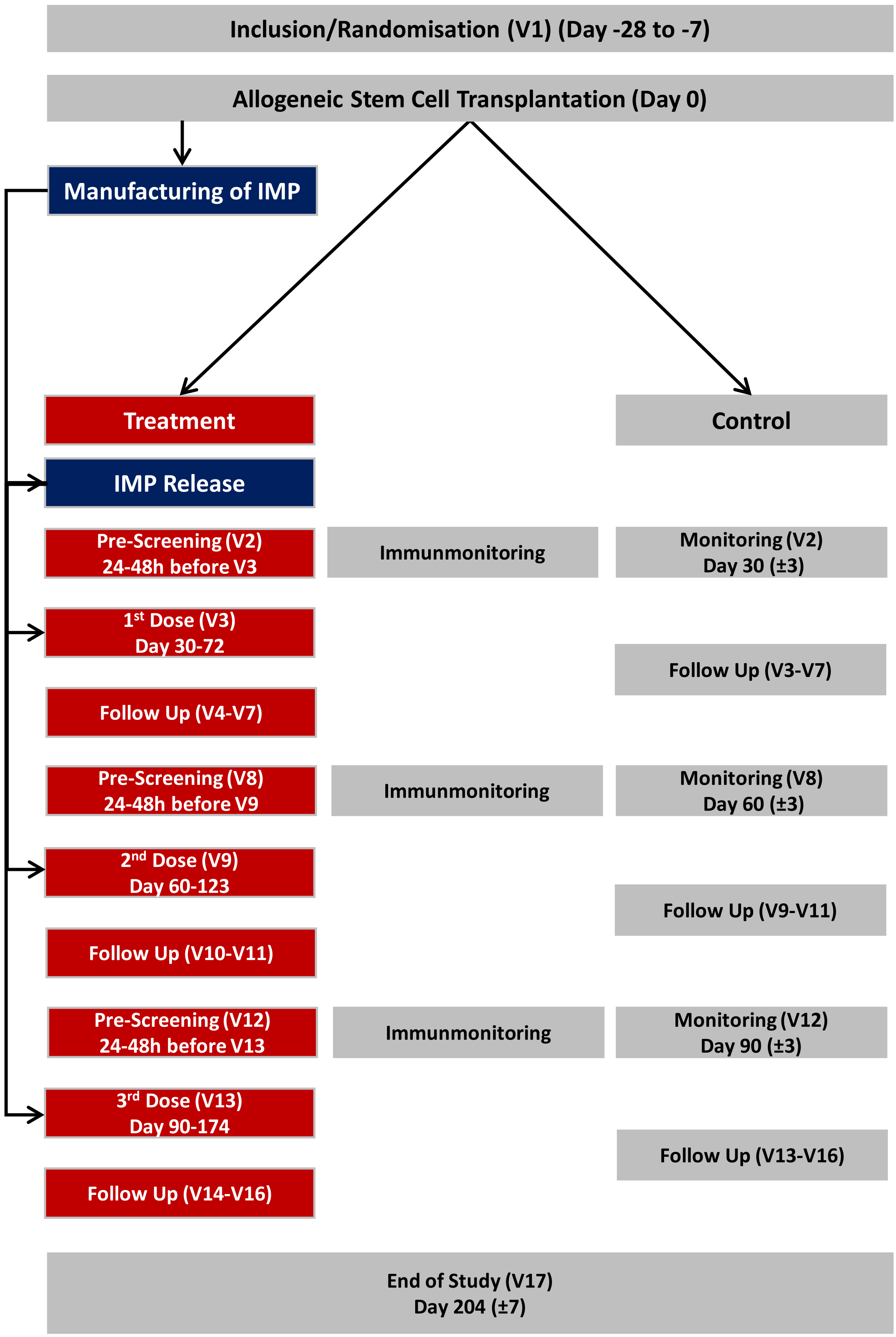

Supplementary Figure 2

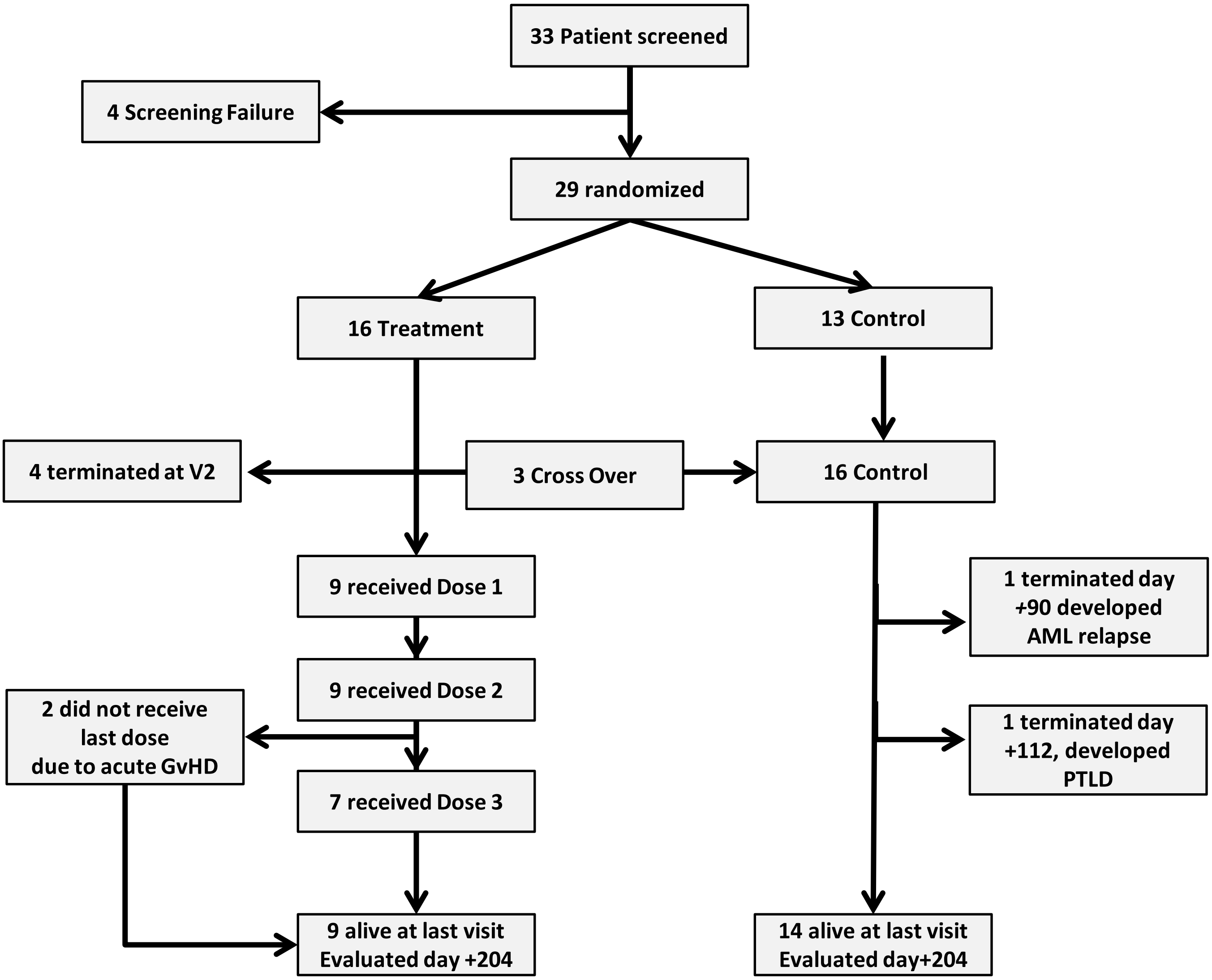

Supplementary Figure 3

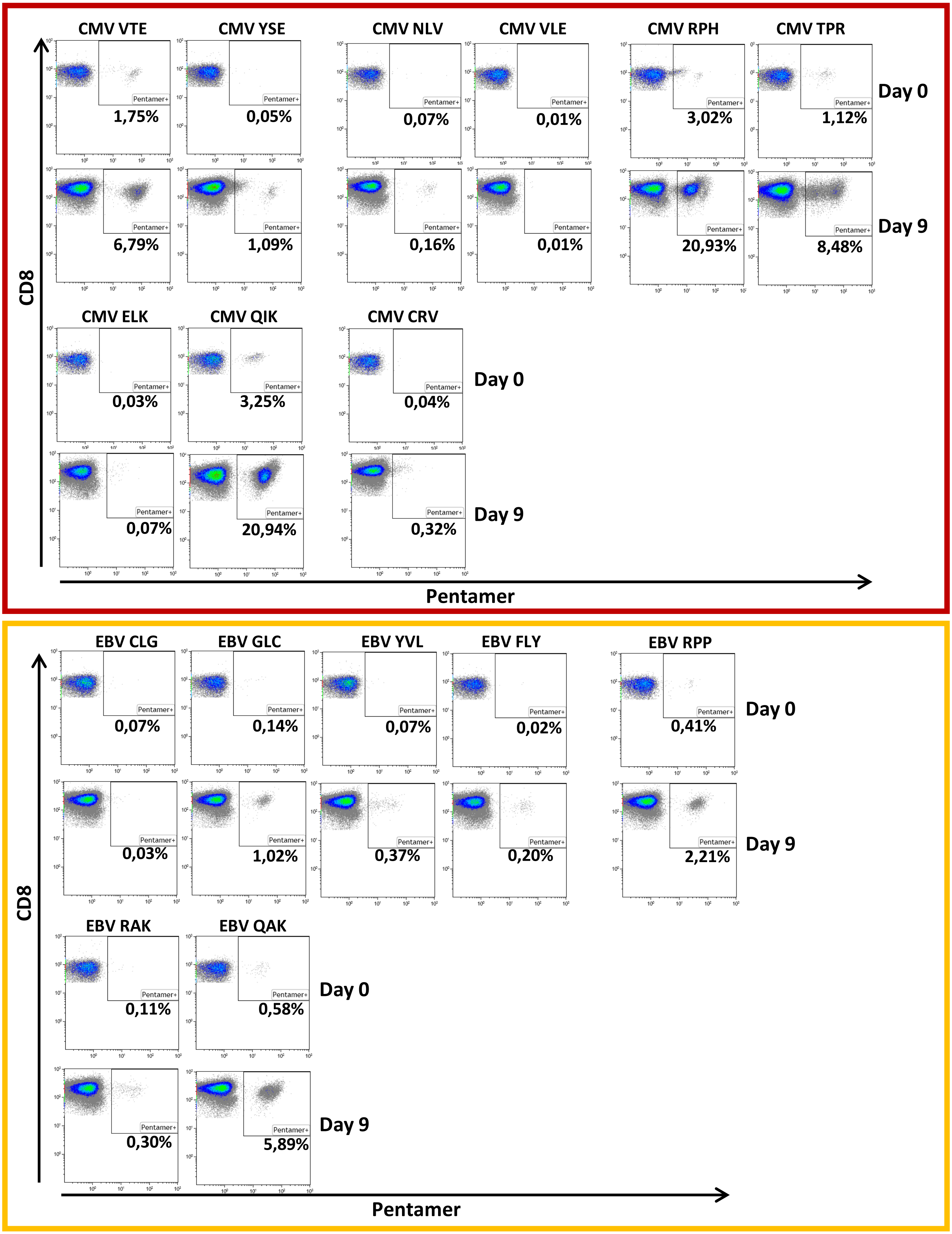

Supplementary Figure 4

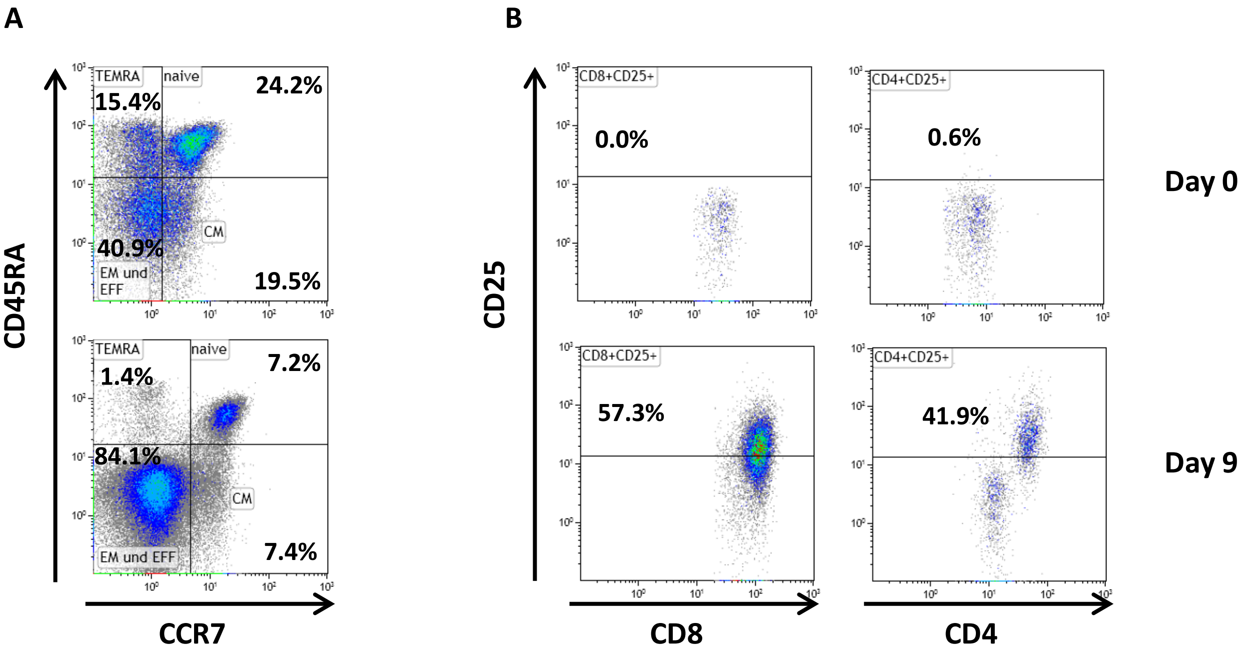

Supplementary Figure 5

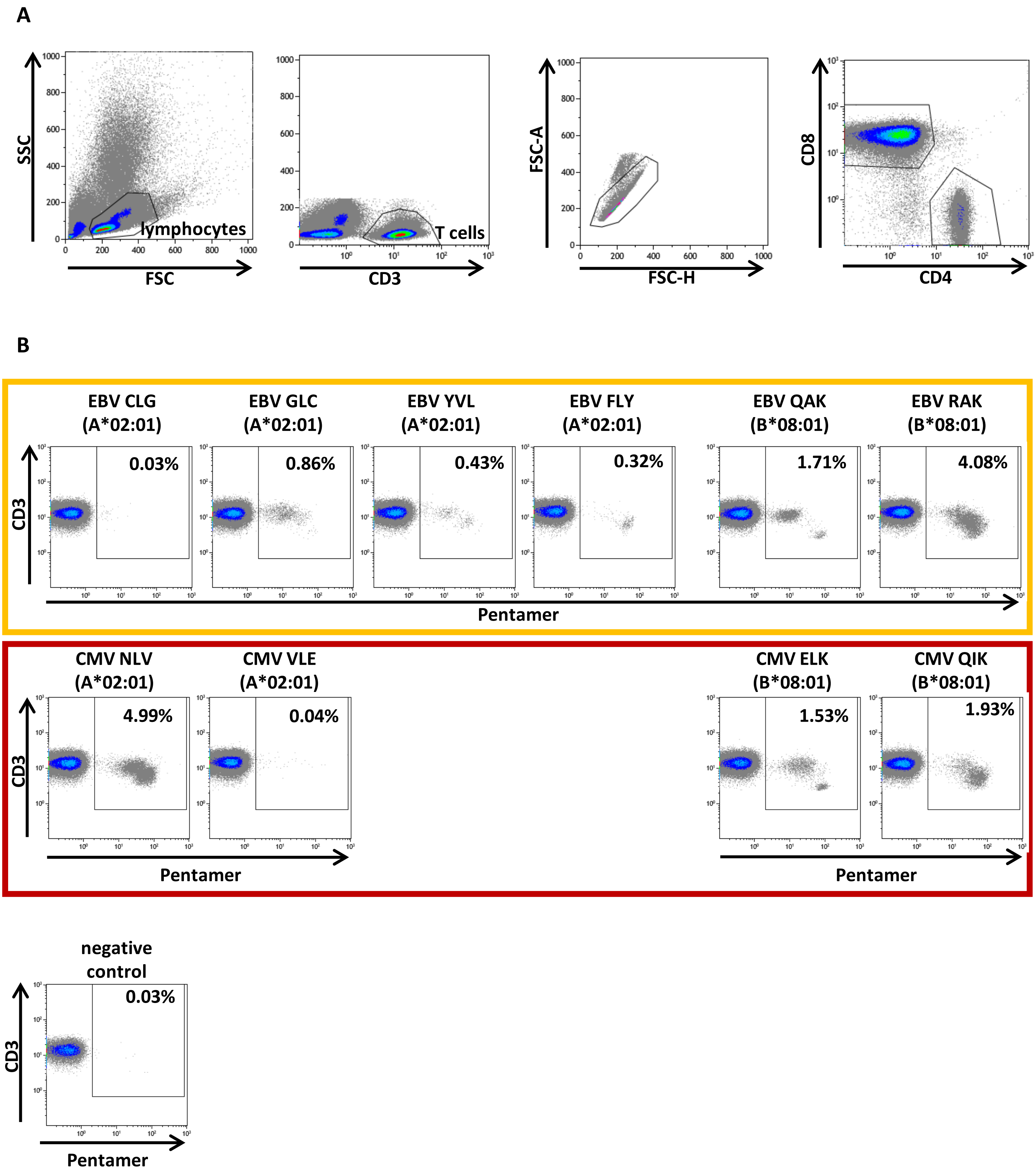

Supplementary Table 1

**Inclusion Criteria**	Male or female between 18 and 75 years of age
	Able to give to understand and consent to the study protocol. Ability to follow guidance by the study personal.
	Written informed consent present after consultation by the study PI or delegate.
	Hematologic or oncologic disease with indication for allogeneic stem cell transplantation
	GCSF mobilized peripheral blood stem cells as stem cell source.
	Presence of at least one HLA gene locus as listed in addendum of the study protocol.
	CMV-serostatus known (positive or negative)
	EBV-serostatus known (positive or negative)
	Female patients: menopausal, or adequate contraception for 12 months
**Inclusion Criteria Donor**	HLA match 10/10, related or unrelated
	EBV-serostatus positive
	CMV-serostatus positive
**Exclusion Criteria Host**	Bone marrow or cord blood as stem cell source.
	Alemtuzumab (Campath®) containing conditioning regimen, or application less than 30 days before stem cell transplantation.
	Clinically relevant comorbidity with Sorror score greater than 3
	Other severe conditions and organ dysfunction (cardiac insufficiency, NYHA III-IV, active Hepatitis B, severe respiratory insufficiency).
	Severe psychological/psychiatric disorder.
	Any other disease or medical condition that is not compatible with participation in the study, after evaluation by the PI.
	Known allergy against any ingredient of the study drug (DMSO).
	Pregnancy or breastfeeding
	Dependency or any relationships between sponsor or PI.
	Planned absence after treatment that interferes with visit schedule.
	Participation in other clinical trials with application of not approved substances.
**Exclusion Criteria Donor**	EBV-serostatus negative
	CMV-serostatus negative
**Additional inclusion criteria before each application of the IMP**	No signs of acute infection (Temp. >38.0C)
	Any other disease or medical condition that is not compatible with participation in the study, after evaluation by the PI.
	No clinical or laboratory evidence for acute GvHD Glucksberg II-IV
	No systemic therapy with prednisolon (or equivalent) >0.5mg/kg bodyweight.
	No steroid refractory GvHD
